# Prevalence of Hearing Impairment by Age: 2nd to 10th Decades of Life

**DOI:** 10.3390/biomedicines10061431

**Published:** 2022-06-17

**Authors:** Koichiro Wasano, Takashi Nakagawa, Kaoru Ogawa

**Affiliations:** 1 Department of Otolaryngology, Head and Neck Surgery, Tokai University School of Medicine, 143 Shimokasuya, Isehara 259-1193, Japan; 2 Division of Hearing and Balance Research, National Institute of Sensory Organs, National Hospital Organization Tokyo Medical Center, 2-5-1 Higashigaoka Meguro, Tokyo 152-8902, Japan; 3 Department of Otorhinolaryngology, Graduate School of Medical Sciences, Kyushu University, Maidashi 3-1-1, Higashi-ku, Fukuoka 812-8582, Japan; nakagawa.takashi.284@m.kyushu-u.ac.jp; 4 Department of Otolaryngology-Head and Neck Surgery, Keio University School of Medicine, 35 Shinanomachi Shinjuku, Tokyo 160-8582, Japan; ogawak@a5.keio.jp

**Keywords:** hearing loss, hearing impairment, prevalence

## Abstract

Background: Accurate data on the prevalence of hearing impairment and severity across age and gender are paramount to formulate hearing health policies. Here, we sought to analyze audiometric data from a large group of age-diverse people in Japan, which has not been previously described in detail. Methods: We analyzed retrospective hearing threshold data of 23,860 participants (10–99 years; left-right hearing threshold difference <15 dB; air-bone gap ≤10 dB) at 500, 1000, 2000, and 4000 Hz, and then classified them for hearing impairment severity according to the WHO Classification. Findings: There was a significant gender difference in median hearing thresholds, starting in 20-year-olds up to early 80-year-olds. Twenty-five percent of men in their late 50s had some level of HI, ~50% in their late 60s, and ~75% in their late 70s. For women, 25% had some level of HI in their early 60s, ~50% in their early 70s, and ~75% in their late 70s. For participants in their early 80s, 50% of either gender had moderate or more severe HI. Interpretation: Our results, derived from a large number of participants, provide basic information about the prevalence of hearing loss by age decade. Since people can expect to live longer than those in previous generations, our detailed data can inform national social systems responsible for hearing screening in making decisions about hearing-aid qualification, which may reduce barriers to older people’s independence, productivity, and quality of life.

## 1. Introduction

The Global Burden of Disease Study [1]—which was funded by the World Health Organization (WHO), Harvard School of Public Health, and the World Bank—reported that an estimated 1·57 billion (95% uncertainty interval 1·51–1·64) people globally had hearing loss in 2019 [2]. The number is presumed to be ever-increasing. The study predicted that nearly 2.5 billion people will have some degree of hearing loss by 2050 and that at least 700 million will require hearing rehabilitation by then. Using the metric years lived with disability (YLDs) to compare morbidity associated with different non-fatal conditions, the authors showed that age-related hearing loss and other causes are the third largest cause of global YLDs in 2019. Moreover, hearing loss is the leading cause of global YLDs for people older than 70 years [2,3].

Another study estimated that, in 2019, the total global economic costs of failing to address hearing loss exceeded USD 981 billion [4]. These costs include other healthcare costs unrelated to hearing loss as well as costs incurred by the local community for education, consequences of the inability to work in certain sectors, and poor quality of life [4].

Recently, reports on early interventions addressing hearing loss have received increasing attention [5,6], because the relationship between untreated hearing loss and dementia and depression is becoming increasingly clear [7,8,9,10,11]. In 2021, Sardone R. et al. reported an association between age-related hearing loss and cognitive frailty, which is associated with multiple adverse health-related outcomes, including falls, mobility loss, disability in activities of daily living, and early mortality [12]. As a result, hearing-aid use has increased in Europe [13]. However, the rate of hearing-aid use remains low in Japan [14]. One reason for this disparity could be the failure of individuals in Japan to acknowledge moderate hearing loss. Patients with severe hearing loss may spontaneously visit an ENT doctor or audiologist to purchase hearing aids, recognizing that their poor hearing creates many problems in daily life. In addition, they may receive encouragement to obtain and use hearing aids, as there is public support for those with severe hearing loss. On the other hand, few patients with mild or moderate hearing loss are likely to visit a doctor and purchase hearing aids, perhaps thinking that ‘my hearing is not so bad because it is almost the same as my friends’ in the same age group’. To reduce this hesitancy and provide those with mild-to-moderate hearing loss with adequate hearing management, some public health intervention is needed. 

Accurate population data on the prevalence of hearing impairment and the rate of minor, moderate, and severe hearing loss across the age spectrum are very important if public health professionals are to formulate national policies about hearing-loss management. However, to date, there are only a few reports about these details [15,16], most likely because it is time-consuming and difficult to accurately perform appropriate hearing tests on a large population of subjects.

In our previous study, we built and analyzed the largest database to date of hearing thresholds and other audiometric data of Japanese people aged 10–90 years [17]. These data were obtained through a retrospective clinical chart cross-sectional study and reported standard age- and gender-specific audiometric data [17]. To avoid bias from outliers, we excluded data of patients who had hearing thresholds that deviated from the population means.

In the present study, we analyzed the entire dataset comprising 23,860 native Japanese-speaking participants, including a re-evaluation of participants’ data that were outliers. The subjects ranged in age from 10 to 99 years. We report the prevalence of hearing impairment and severity by age decade.

## 2. Materials and Methods

### 2.1. Study Design and Data Collection

We conducted a retrospective cross-sectional study of a broad age range of patients in Japan. A total of 30,147 patients and 69,222 associated pure-tone audiometry tests were available to be evaluated for inclusion. Audiometric data of some of these participants were presented in a previous report [17]. To be eligible for inclusion in the present study, patients had to be native-Japanese speakers between the ages of 10 and 99 years and had to have visited the Department of Otolaryngology at National Hospital Organization Tokyo Medical Center between April 2000 and March 2020; patients were seeking medical attention for various reasons. We collected patients’ age at the time of audiometric testing. The incidence of symmetrical sensorineural hearing loss caused by some diseases other than the age-related and congenital hearing loss is low. Nonetheless, to ensure that patients with those kinds of hearing losses were not included in our present analyses, we excluded patients who had a left-right ear hearing-threshold differences of ≥15 dB at 250, 500, 1000, 2000, and 4000 Hz and had an air-bone gap of >10 dB. Patient audiometric and demographic data were obtained from electronic medical records, as described earlier [16]. If a patient underwent pure-tone audiometry multiple times, the mean age at each test and mean hearing threshold for each frequency were used.

This retrospective study was approved by the Tokyo Medical Center’s Institutional Review Board (IRB No. R20-087). Written informed consent was not obtained because of the study’s retrospective design and because it was impracticable. Included patients’ personal details and their data were de-identified.

### 2.2. Classification of Hearing Loss Severity

For the main analyses, we calculated the simple arithmetic mean hearing threshold, according to the method described in the WHO International Classification of Impairments, Disabilities, and Handicaps [18]. Briefly, the hearing thresholds at 500, 1000, 2000, and 4000 Hz were summed and then divided by 4 to obtain the pure-tone average of each participant (Table 1). First, we analyzed the distribution of hearing loss severity by age decade. We used the Shapiro–Wilk statistical test to assess whether the distribution of thresholds for each age decade was normal. The Mann–Whitney test was used to evaluate the differences between males and females. Violin plots were constructed to quantify and visually depict the age- and gender-specific distribution of mean hearing thresholds.

To classify hearing loss severity, we graded hearing thresholds as described in the WHO International Classification of Impairments, Disabilities, and Handicaps [18]. This grading method is based on a scale of 0 to 4, with higher values corresponding to greater severity of hearing loss. The following categories are used: Grade 0 (no impairment), hearing level is ≤25 dB; Grade 1 (slight impairment), hearing level is between 26 dB and 40 dB; Grade 2 (moderate impairment), hearing level is between 41 dB and 60 dB; Grade 3 (severe impairment), hearing level is between 61 dB and 80 dB; Grade 4 (profound impairment including deafness), hearing level is >81 dB. 

We also measured and classified hearing loss according to methods outlined by Japanese law. In Japan, hearing loss is considered to be a public health issue; thus, Japan has enacted various laws and acts that take hearing into account. Those laws also describe methods for calculating and classifying hearing loss. For example, the Law for the Welfare of Physically Disabled Persons stipulates that hearing loss is to be assessed at 1000 Hz and that a weighted average is to be used to determine whether an individual qualifies for welfare support due to hearing impairment [19]. On the other hand, the Labor Standards Act stipulates that hearing should be assessed at middle range frequencies and that a weighted average is used for workers’ compensation cases (Table 1) [20]. For these Japanese laws, the classification of hearing loss severity is based on the classification system of the Japan Audiological Society [21]: Mild hearing loss—hearing threshold is between 25 dB and 40 dB; moderate hearing loss—hearing threshold is between 40 dB and 70 dB; severe hearing loss—hearing threshold is ≥70 dB. While most Japanese laws adhere to those standards, the Japanese Health Insurance Act defines severe hearing loss as hearing thresholds of 60 dB or higher [22]. As interventions and measures against hearing loss are a public health concern, we also applied the calculation and classification methods prescribed by Japanese law to our data set (Table 1) and performed additional analysis on those data accordingly.

### 2.3. Statistical Analysis

We used the Shapiro–Wilk statistical test to assess whether the distribution was normal. As all parameters in our study were distributed non-normally, the Mann–Whitney test was used to evaluate differences. All statistical analyses were performed using GraphPad Prism 9.2.0 (GraphPad Software, San Diego, CA, USA). *p* < 0.05 was considered statistically significant. GraphPad was also used to construct all graphs and visual depictions of data.

### 2.4. Role of the Funding Source

The funder of the study had no role in study design, data collection, data analysis, data interpretation, or writing of the report.

## 3. Results

**Figure 1:**
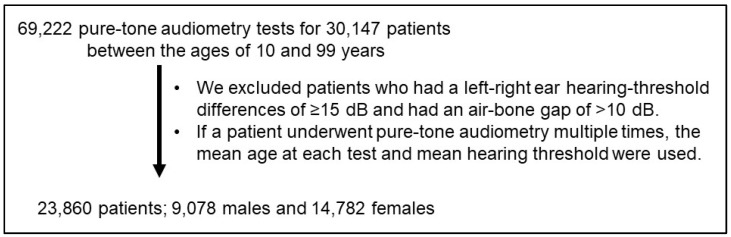
Study design and the number of subjects.

We identified 23,860 patients (Table 2) who met our inclusion criteria (Figure 1). In general, more participants were female in all age brackets. Although ~75% of participants were between the ages of 30 and 79 years old, about 14% of participants were older than 80 years old, with almost 400 in the 90–99-year age bracket.

**Figure 2:**
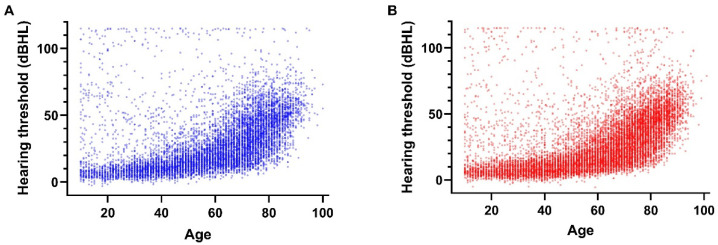
Gender- and age-specific scatter plots of mean hearing thresholds (N = 23,860). (**A**) Males (N = 9078). (**B**) Females (N = 14,782). Average of left- and right-side hearing thresholds was calculated for each subject at 500, 1000, 2000, and 4000 Hz, and then the simple arithmetic mean over the four frequencies for each subject was calculated and plotted. Some subjects’ mean threshold exceeded the ceiling dBHL of 110 dBHL, in which case they were assigned a dBHL value of 115.

**Figure 3:**
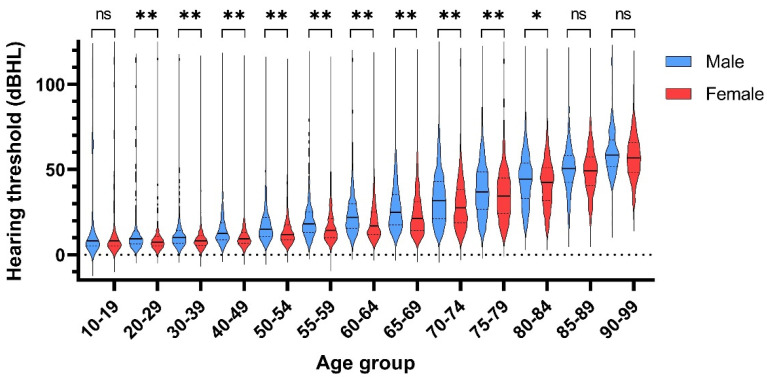
Gender- and age-specific violin plots of hearing thresholds. Hearing thresholds increased with age, and there were significant differences between males and females from their 20s to early 80s. The upper-dashed, solid, and lower-dashed horizontal lines in each dispersion envelope indicate the 75 percentile, the median, and the 25 percentile, respectively. The widths of the violin plots depict distribution density of hearing thresholds. The medium level of smoothing was used in GraphPad for the distribution density. *: *p* < 0.05, **: *p* < 0.01, ns: not significant.

The gender-specific distributions of average hearing thresholds are shown in Figure 1. For all age decades, regardless of gender, the distribution of the average hearing threshold was non-normal, being skewed towards higher thresholds (Table 3). Within-age-group variability (data spread) of average hearing thresholds also increased with increasing age (Figure 2). The median hearing threshold also increased with age, and there was a significant difference in hearing thresholds between males and females in each decade from the 20s to early 80s with small to medium effect sizes (Figure 3, Table S1).

**Figure 4:**
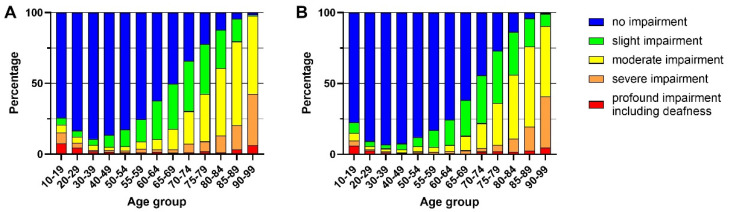
Percentage of participants in each hearing-loss severity, in the five WHO grades [18]. (**A**) Males; (**B**) females.

The percentages of participants in the five WHO grades (Grades 0–4) [18] of hearing loss severity are shown in Figure 4 and Table S1. Twenty-five percent of men in their late 50s had some level of hearing impairment (Grade 1, 2, 3, or 4), while 50% of men in their late 60s and 75% in their late 70s had some level of hearing impairment (Grade 1, 2, 3, or 4). For men in their early 80s, more than 50% had moderate or severe hearing impairment (Grade 3 or 4, respectively). In general, the percentage of men or women with severe and profound hearing impairment accelerated with age. For men in their 90s, only a small percentage had no impairment or slight impairment (Grade 0 or 1, respectively).

A similar pattern of hearing loss severity was observed in female participants across the age spectrum, but the age onset appeared to be later for women. Twenty-five percent of women in their late 60s had some level of hearing impairment (Grade 1, 2, 3, or 4), while 50% of women in their early 70s and 75% in their late 70s had some level of hearing impairment (Grade 1, 2, 3, or 4). As with men, more than 50% of women had moderate or severe hearing loss in their early 80s. For women in their 90s, about 10% had a slight impairment (Grade 1).

The results from using the methods for assessing hearing loss outlined by Japanese law are presented in Tables S2–S4. 

## 4. Discussion

Hearing impairment is a significant health burden globally [2], one that also has many other indirect health and social consequences [4]. It is predicted that nearly 2.5 billion people will have some degree of hearing loss by 2050 and that at least 700 million will require hearing rehabilitation by then [2]. However, hearing-aid use in some countries remains low and, to date, there are only a few studies that report large-scale prevalence data on the degree of mild, moderate, and severe hearing loss in a wide spectrum of ages, especially in the oldest population [23]. This knowledge gap presents a problem when trying to formulate rational social and health-management policies for hearing impaired patients and others seeking treatment. In the present study, we report the prevalence and severity of hearing impairment by age decade, using retrospective clinical audiometric records of 23,860 participants. By excluding subjects whose hearing thresholds had a large right-left difference or subjects who had an air-bone gap > 10 dB, we ensured that the results were based on data that were obtained almost entirely from subjects who had congenital or early-onset hearing loss or age-related hearing loss. These prevalence data will be valuable for formulating public policy.

In the present study, the prevalence of hearing loss in patients visiting our clinic decreased from the second decade through to the fourth decade (10s to 30s years). As a majority of congenital or early-onset hearing loss can be detected by neonatal screening [24], patients in these age brackets tend to seek medical treatment less frequently for hearing issues. This phenomenon might partially explain why our prevalence data showed that only a small percentage of participants had any hearing impairment as defined by the WHO classification (Figure 3). After the fourth decade, we recorded an increase in the prevalence of hearing impairment, which may reflect the onset of age-related hearing loss.

In each decade from the 20s to early 80s, the median hearing thresholds in men were significantly higher than in women. It is consistent with the past reports and was reported to be attributable to genetic and environmental factors [15,25,26]. As the hearing threshold shift caused by environmental factors such as noise exposure and tobacco smoking could be prevented, the contribution rate of each environmental factor is desirable to be elucidated by further studies.

One international health tracking survey on subjective hearing impairment and hearing-aid adoption showed that there was no difference in the prevalence of self-reported hearing loss rates between Europe [12] and Japan [13], which were 10.6% and 11.3%, respectively. However, the hearing-aid adoption rate for the hearing impaired was significantly different, being 41.6% in Europe versus 14.4% in Japan. Although the reason why the adoption rate is lower in Japan is not fully determined, it might be caused by the social awareness and doctors’ attitudes toward hearing impairment and hearing-aid adoption. In order to appropriately manage hearing impairment rehabilitation, an awareness campaign is important and hearing screening procedures for older adults should be the same as newborn hearing screening procedures [27]. 

A leader in this approach is Singapore. In 2018, the Singapore government launched Project Silver Screen for citizens aged 60 and above, which screens for vision, oral, and hearing problems [28]. Hearing screening is performed via two kinds of questionnaires that assess hearing loss and tinnitus, checking of the ear canals using an otoscope, and pure-tone audiometry testing. Research on the relationship between these aural problems and falling were emphasized as a motivation for hearing-loss screening [29,30].

For a relatively small nation as Singapore, the screening of all citizens aged 60 and above is reasonable, because its population is relatively small at 5.45 million [31]. For countries with larger populations, like Japan (125 million) [32], it is prohibitive to screen all citizens because of the high cost and excessive time commitment. Thus, an age- and gender-specific analysis of prevalence from population data may provide some guidance for formulating policy in the absence of widespread blanket screening.

Our analysis has limitations. First, the study sample is based on retrospectively collected data from chart records of patients who underwent pure-tone audiometry for various reasons in the otolaryngology department at a major general hospital in Tokyo. Therefore, it might include some cases with hearing loss considered to be more severe than what is observed normally in a population of people not seeking medical attention. However, it is very difficult to evaluate thousands of people—from young people to the elderly—with high-quality pure-tone audiometry without any tendency. Therefore, our results can be considered to provide basic information about the prevalence of hearing loss by age bracket. Second, the data were collected over 20 years, and the analysis is a secondary analysis of data collected for a different purpose. Nonetheless, this is an effective means of research, as primary data collection is too costly or infeasible. Although our department has standard procedures for performing audiometric testing, we cannot be certain that the data might not have been collected systematically and that there might have been slight differences in data collection over the 20 years of audiometric testing.

In conclusion, based on the results of the present study, population-wide hearing-loss screening initiatives should target those over 70 years old. This recommendation seems warranted since more than one-half of our subject population in these age groups had some level of hearing impairment. For more focused screening initiatives, the threshold for beginning routine screening should begin after age 80, since our present findings showed that more than one-half of those 80 years and older have moderate or severe hearing impairment. Public or social initiatives for hearing-loss screening should also direct individuals to where and how they can obtain more in-depth audiometric examination to promote hearing-aid adoption [33]. Increasing the hearing-aid adoption rate may be a way to extend older adults’ independence [33] and could go a long way towards reducing the incidence of dementia and depression, which is becoming increasingly more common in older people [7,8,9,10,11]. 

## Figures and Tables

**Table 1:** Method for calculating average hearing threshold from individual pure-tone audiometry tests at four frequencies.

A: Average of left- and right-side hearing thresholds assessed at 500 Hz
B: Average of left- and right-side hearing thresholds assessed at 1000 Hz
C: Average of left- and right-side hearing thresholds assessed at 2000 Hz
D: Average of left- and right-side hearing thresholds assessed at 4000 Hz
Simple arithmetic mean = (A + B + C + D)/4
1000 Hz weighted mean = (A + 2B + C)/4
Middle range weighted mean = (A + 2B + 2C + D)/6

**Table 2:** Age- and gender-specific distribution of participants *.

Age (Years)	Male N (%)	Female N (%)	Total N (%)
10–19	405 (4.5)	530 (3.6)	935 (3.9)
20–29	521 (5.8)	1043 (7.1)	1564 (6.6)
30–39	868 (9.6)	1537 (10.4)	2405 (10.1)
40–49	1068 (11.8)	1739 (11.8)	2807 (11.8)
50–54	603 (6.6)	979 (6.6)	1582 (6.6)
55–59	664 (7.3)	1068 (7.2)	1732 (7.3)
60–64	834 (9.2)	1244 (8.4)	2078 (8.7)
65–69	933 (10.3)	1446 (9.8)	2379 (10.0)
70–74	990 (10.9)	1563 (10.6)	2553 (10.7)
75–79	969 (10.7)	1502 (10.2)	2471 (10.4)
80–84	695 (7.7)	1180 (8.0)	1875 (7.9)
85–89	387 (4.3)	699 (4.7)	1086 (4.6)
90–99	141 (1.6)	252 (1.7)	393 (1.6)
Totals	9078 (38.0)	14,782 (62.0)	23,860

* Included participants had a left-right ear hearing-threshold difference of ≤15 dB at 250, 500, 1000, 2000, and 4000 Hz and had an air-bone gap of <10 dB. For some age brackets, we show the data by lustrum or 5-year bins.

**Table 3:** Results of the Shapiro–Wilk test for assessing the normality of hearing threshold distributions.

		Age Group												
		10–19	20–29	30–39	40–49	50–54	55–59	60–64	65–69	70–74	75–79	80–84	85–89	90–99
Male	W *	0.7	0.59	0.55	0.62	0.7	0.74	0.8	0.88	0.94	0.97	0.99	0.96	0.93
	*p*-value	<0.01	<0.01	<0.01	<0.01	<0.01	<0.01	<0.01	<0.01	<0.01	<0.01	<0.01	<0.01	<0.01
Female	W *	0.65	0.48	0.5	0.57	0.68	0.75	0.78	0.87	0.88	0.93	0.96	0.97	0.99
	*p*-value	<0.01	<0.01	<0.01	<0.01	<0.01	<0.01	<0.01	<0.01	<0.01	<0.01	<0.01	<0.01	0.041

* Value of the Shapiro–Wilk test.

## Data Availability

Will individual participant data be available? Yes. What data in particular will be shared? Individual participant data that underlie the results reported in this article, after de-identification (text, tables, figures, and appendices). What other documents will be available? Study protocol. When will data be available (start and end dates)? Beginning 3 months and ending 5 years following article publication. With whom? Researchers who provide a methodologically sound proposal. For what types of analyses? To achieve the aims in the approved proposal. By what mechanism will data be made available? Proposals and data should be directed to wasano@a5.keio.jp; to gain access, data requestors will need to sign a data access agreement.

## Supplementary Materials

The following supporting information can be downloaded at: https://www.mdpi.com/article/10.3390/biomedicines10061431/s1. Table S1: Distribution of participants according to the four WHO grades of hearing loss severity. Table S2: Distribution of participants according to the classification method of the Japan Audiological Society and the Japanese Health Insurance Act. Table S3: Distribution of participants according to the Law for the Welfare of Physically Disabled Persons in Japan based on 1000 Hz-weighted average. Table S4: Distribution of participants according to the Labor Standards Act in Japan based on middle-range weighted average.
